# Supplementary material for: Comprehensive Evaluation of Immune-Checkpoint DNA Cancer Vaccines in a Rat Cholangiocarcinoma Model
Source: Vaccines (Basel). 2020 Nov 24;8(4):703. doi: 10.3390/vaccines8040703 (PMC7712087; doi:10.3390/vaccines8040703)
Supplement: Supplementary file 1 [file vaccines-08-00703-s001.pdf]

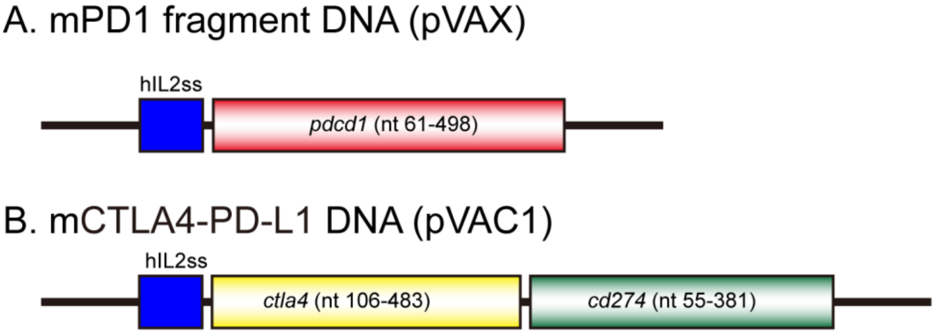

C

	**Mus musculus** *pdcd1* nt 61-498 (GeneID: 18566)	**Mus musculus** *cd274* nt 55-381 (GeneID: 60533)	**Mus musculus** *ctla4* nt 106-483 (GeneID: 12477)
**Rattus norvegicus** *pdcd1* nt 61-498 (GeneID: 301626)	**93%**		
**Rattus norvegicus** *cd274* nt 55-381 (GeneID: 499342)		**93%**	
**Rattus norvegicus** *ctla4* nt 106-483 (Gene ID: 63835)			**94%**

Figure S1. Schema for experimental design for the construct of mPD-1 DNA vaccine. (**A**) Schema for experimental design for the construct of mCTLA4-PD-L1 DNA vaccine, (**B**) The similarities of PD1, PD-L1, and CTLA4 nucleotide sequences between mice (*Mus musculus*) and rats (*Rattus norvegicus*).

```
Mus musculus pdcd1 (nt 61-498) and Rattus norvegicus pdcd1 (nt 61-498)
Score:614 bits(332), Expect:2e-180,
Identities:409/438(93%),  Gaps:0/449(0%), Strand: Plus/Plus

Mus musculus         1    TCAGGTTGGTTGCTAGAGGTCCCCAATGGGCCCTGGAGGTCCCTCACCTTCTACCCAGCC   60
                          ||||| ||| | |||||||||| ||||  |||||||||| |||||||||||| |||| ||
Rattus norvegicus    1    TCAGGGTGGCTTCTAGAGGTCCTCAATAAGCCCTGGAGGCCCCTCACCTTCTCCCCAACC   60

Mus musculus         61   TGGCTCACAGTGTCAGAGGGAGCAAATGCCACCTTCACCTGCAGCTTGTCCAACTGGTCG   120
                          |||||||||||||||||||||||||||||||||||||||||||| || ||||||||||||
Rattus norvegicus    61   TGGCTCACAGTGTCAGAGGGAGCAAATGCCACCTTCACCTGCAGTTTCTCCAACTGGTCG   120

Mus musculus         121  GAGGATCTTATGCTGAACTGGAACCGCCTGAGTCCCAGCAACCAGACTGAAAAACAGGCC   180
                          |||||||||| |||||||||| |||| |||||||||||||||||||||||||||||||||
Rattus norvegicus    121  GAGGATCTTAAGCTGAACTGGTACCGTCTGAGTCCCAGCAACCAGACTGAAAAACAGGCC   180

Mus musculus         181  GCCTTCTGTAATGGTTTGAGCCAACCCGTCCAGGATGCCCGCTTCCAGATCATACAGCTG   240
                          |||||||| |||||||  ||||| ||||||| ||||||||||||||||||| |||| |||
Rattus norvegicus    181  GCCTTCTGCAATGGTTACAGCCAGCCCGTCCGGGATGCCCGCTTCCAGATCGTACAACTG   240

Mus musculus         241  CCCAACAGGCATGACTTCCACATGAACATCCTTGACACACGGCGCAATGACAGTGGCATC   300
                          |||||| | ||||||||||||||||||||||| ||  |||||||||||||||||||||||
Rattus norvegicus    241  CCCAACGGACATGACTTCCACATGAACATCCTCGATGCACGGCGCAATGACAGTGGCATC   300

Mus musculus         301  TACCTCTGTGGGGCCATCTCCCTGCACCCCAAGGCAAAAATCGAGGAGAGCCCTGGAGCA   360
                          |||||||||||||||||||||||||  ||||||||| ||||| | ||||| |||||||||
Rattus norvegicus    301  TACCTCTGTGGGGCCATCTCCCTGCCTCCCAAGGCACAAATCAAAGAGAGTCCTGGAGCA   360

Mus musculus         361  GAGCTCGTGGTAACAGAGAGAATCCTGGAGACCTCAACAAGATATCCCAGCCCCTCGCCC   420
                          ||||| ||||||||||||||||||||||||||| |||||||||||||||| ||||| |||
Rattus norvegicus    361  GAGCTTGTGGTAACAGAGAGAATCCTGGAGACCCCAACAAGATATCCCAGACCCTCACCC   420

Mus musculus         421  AAACCAGAAGGCCGGTTT   438
                          || |||||||||| ||||
Rattus norvegicus    421  AAGCCAGAAGGCCAGTTT   438
```

Figure S2. A nucleotide sequence alignment of mouse *pdcd1* and rat *pdcd1*.

Mus musculu *cd274* (nt 55-381) and Rattus norvegicus *cd274* (nt 55-381)

Score: 486 bits(263), Expect: 3e-142,

Identities: 305/327(93%), Gaps:0/326(0%), Strand: Plus/Plus

```
Mus musculus        1     TTTACTATCACGGCTCCAAAGGACTTGTACGTGGTGGAGTATGGCAGCAACGTCACGATG  60
                          |||||||||||||||||||||||| |||||||||||||||||||||||||||||||||||
Rattus norvegicus   1     TTTACTATCACGGCTCCAAAGGACCTGTACGTGGTGGAGTATGGCAGCAACGTCACGATG  60

Mus musculus        61    GAGTGCAGATTCCCTGTAGAACGGGAGCTGGACCTGCTTGCGTTAGTGGTGTACTGGGAA  120
                          |||||||||||||||||||||| |||||||||||||| ||| ||||||||||||||||||
Rattus norvegicus   61    GAGTGCAGATTCCCTGTAGAACAGGAGCTGGACCTGCATGCATTAGTGGTGTACTGGGAA  120

Mus musculus        121   AAGGAAGATGAGCAAGTGATTCAGTTTGTGGCAGGAGAGGAGGACCTTAAGCCTCAGCAC  180
                          ||||||||  |||||||||||||| ||||||| |||||||||||| | ||||||||||||
Rattus norvegicus   121   AAGGAAGACAAGCAAGTGATTCAGCTTGTGGCGGGAGAGGAGGACATAAAGCCTCAGCAC  180

Mus musculus        181   AGCAACTTCAGGGGGAGAGCCTCGCTGCCAAAGGACCAGCTTTTGAAGGGAAATGCTGCC  240
                          || | ||||||||||||||||||  ||||||||||||||||| |||||||||||||||| 
Rattus norvegicus   181   AGTAGCTTCAGGGGGAGAGCCTCCTTGCCAAAGGACCAGCTTCTGAAGGGAAATGCTGCA  240

Mus musculus        241   CTTCAGATCACAGACGTCAAGCTGCAGGACGCAGGCGTTTACTGCTGCATAATCAGCTAC  300
                          ||||||||||| || ||||||||||||||||||||||| ||||||||||| |||||||||
Rattus norvegicus   241   CTTCAGATCACGGATGTCAAGCTGCAGGACGCAGGCGTCTACTGCTGCATGATCAGCTAC  300

Mus musculus        301   GGTGGTGCGGACTACAAGCGAATCACG  327
                          || ||||||||||||||||||||||| 
Rattus norvegicus   301   GGCGGTGCGGACTACAAGCGAATCACA  327
```

Figure S3. A nucleotide sequence alignment of mouse *cd274* and rat *cd274.*

Mus musculus *ctla4* (nt 106-483) and Rattus norvegicus *ctla4* (nt 106-483)

Score: 577 bits(312), Expect:2e-169,

Identities: 356/378(94%), Gaps: 0/378(0%), Strand: Plus/Plus

```
Mus musculus         1 GAAGCCATACAGGTGACCCAACCTTCAGTGGTGTTGGCTAGCAGCCATGGTGTCGCCAGC  60
                       ||||||||||| ||||||||||||||||||||||||||| ||||||| ||||||||||||
Rattus norvegicus    1 GAAGCCATACAAGTGACCCAACCTTCAGTGGTGTTGGCCAGCAGCCACGGTGTCGCCAGC  60

Mus musculus        61 TTTCCATGTGAATATTCACCGTCACACAACACTGATGAGGTCCGGGTGACTGTGCTGCGG  120
                       ||||||||||||||| || | ||||||||||||||||||||||||||||| |||||||||
Rattus norvegicus   61 TTTCCATGTGAATATGCATCTTCACACAACACTGATGAGGTCCGGGTGACGGTGCTGCGG  120

Mus musculus       121 CAGACAAATGACCAAATGACTGAGGTCTGTGCCACGACATTCACAGAGAAGAATACAGTG  180
                       ||||||||||||||| |||| ||||||||||||||||||||||||| |||||| ||  ||
Rattus norvegicus  121 CAGACAAATGACCAAGTGACAGAGGTCTGTGCCACGACATTCACAGTGAAGAACACGTTG  180

Mus musculus       181 GGCTTCCTAGATTACCCCTTCTGCAGTGGTACCTTTAATGAAAGCAGAGTGAACCTCACC  240
                       |||||||||||| |||||||||||||||||||||||||||||||||||||||||||||||
Rattus norvegicus  181 GGCTTCCTAGATGACCCCTTCTGCAGTGGTACCTTTAATGAAAGCAGAGTGAACCTCACC  240

Mus musculus       241 ATCCAAGGACTGAGAGCTGTTGACACGGGACTGTACCTCTGCAAGGTGGAACTCATGTAC  300
                       |||||||||||||| |||| |||||| ||||||||| |||||||||||||||||||||||
Rattus norvegicus  241 ATCCAAGGACTGAGGGCTGCTGACACCGGACTGTACTTCTGCAAGGTGGAACTCATGTAC  300

Mus musculus       301 CCACCGCCATACTTTGTGGGCATGGGCAACGGGACGCAGATTTATGTCATTGATCCAGAA  360
                       ||||||||||||||||||||||||||||||||||| |||||||||||||| |||||||||
Rattus norvegicus  301 CCACCGCCATACTTTGTGGGCATGGGCAACGGGACCCAGATTTATGTCATCGATCCAGAA  360

Mus musculus       361 CCATGCCCGGATTCTGAC  378
                       |||||||| ||||| |||
Rattus norvegicus  361 CCATGCCCAGATTCAGAC  378
```

Figure S4. A nucleotide sequence alignment of mouse *ctla4* and rat *ctla4.*

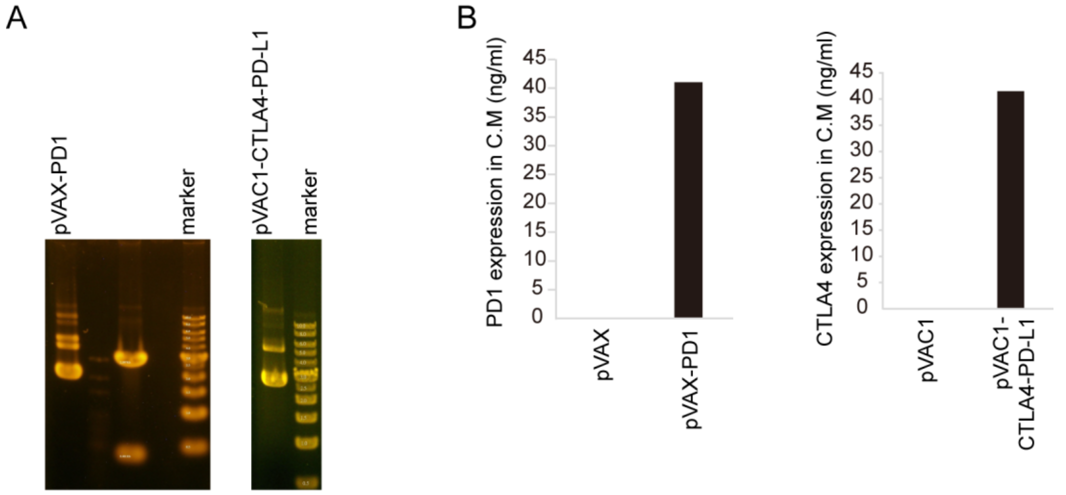

Figure S5. The quality control of DNA vaccines. (**A**) The agarose gel electrophoresis of two plasmids, (**B**) The expression of PD1 or CTLA4 in conditioned medium (C.M.) from CHO cells transiently expressing CTLA4-PDL1 or PD1 for 48 hours.
